# Collapse and recovery of livestock systems shape fire regimes on the Eurasian steppe: a review of ecosystem and biodiversity implications

**DOI:** 10.1098/rstb.2024.0062

**Published:** 2025-04-17

**Authors:** Johannes Kamp, Tejas Bhagwat, Norbert Hölzel, Ilya Smelansky

**Affiliations:** ^1^ Department of Conservation Biology, University of Göttingen, Göttingen 37073, Germany; ^2^ Institute of Landscape Ecology, University of Münster, Münster 48149, Germany; ^3^ Association for the Conservation of Biodiversity of Kazakhstan (ACBK), Astana 010000, Kazakhstan

**Keywords:** burnt area, fire frequency, pyrodiversity, restoration, rewilding, burrowing mammals

## Abstract

Shifts in fire regimes can trigger rapid changes in ecosystem functioning and biodiversity. We synthesize evidence for patterns, causes and consequences of recent change in fire regimes across the Eurasian steppes, a neglected global fire hotspot. Political and economic turmoil following the break-up of the Soviet Union in 1991 triggered abrupt land abandonment over millions of hectares and a collapse of livestock populations. The build-up of vegetation as fuel, rural depopulation and deteriorating fire control led to a rapid increase in fire size, area burned and fire frequency. Fire regimes were also driven by drought, but likely only after fuel had accumulated. Increased fire disturbance resulted in grass encroachment, vegetation homogenization and decreasing plant species diversity. Feedback loops due to the high grass flammability were likely. Direct and carry-on effects on birds, keystone small mammals and insects were largely negative. Nutrient cycling and carbon balance changed, but these changes have yet to be quantified. The regime of large and frequent fires persisted until *ca* 2010 but shifted back to a more grazing-controlled regime as livestock populations recovered, reinforced by increasing precipitation. Key future research topics include the effects of future climate change, changing pyrodiversity and pyric herbivory on ecosystem resilience. Ongoing steppe restoration and rewilding efforts, and integrated fire management will benefit from a better understanding of fire regimes.

This article is part of the theme issue ‘Novel fire regimes under climate changes and human influences: impacts, ecosystem responses and feedbacks’.

## 1. Introduction

Fire is an important driver of the evolution, structure and functioning of ecosystems [1] and their biodiversity [2]. In the Anthropocene, fire regimes, i.e. the timing, location, frequency and size of fires, are changing [3,4]. Fire as a disturbance agent has become more intense and widespread due to human activity, including in ecosystems that were historically neither fire-prone nor fire-adapted [5]. Climate change modulates ignition and fuel flammability [6,7]. Land-use change drives changes in fuel availability, with declines in fuel, e.g. through increasing livestock grazing pressure, and increases where land is abandoned [8]. These processes contribute to abrupt changes in fire regimes [9], including their intensification in some parts of the world [10] and a decrease in fire disturbance in others [3,4].

Wildfires can trigger feedback loops (e.g. via fire-related greenhouse gas emissions, alteration of carbon stocks and changed land surface albedo) that accelerate climate change [11]. Changing fire regimes have important implications for biodiversity, ecosystem resilience and functioning, as well as human livelihoods. It is important to understand fire regime shifts to develop policies for human adaptation, climate change mitigation and biodiversity conservation [3].

Recent advances in mapping changing fire regimes, and their implications for humans, ecosystems and biodiversity, were largely concentrated in fire-prone systems, such as the Mediterranean or savannas in Africa and Australia. Conceptual advances were often also tested in very restricted areas despite their global importance in changing socio-ecological systems. An example is ‘pyric herbivory’, i.e. interactions between fire and grazing by wild and domestic ungulates [12].

As most grasslands globally, the Eurasian steppes, grasslands stretching from eastern Ukraine to the Altai mountains over a belt of 4000 km, have evolved and been shaped by grazing and fire [13]. They are one of the ‘hottest fire hotspots’ globally, as fires are frequent, intense and large compared with other global hotspots [3,14]. Fire regimes were partly shaped by humans over at least the past 1000 years in the steppe [15,16], and there is evidence for fire as a tool for pasture management by nomadic tribes since the eighteenth century [17]. However, fire disturbance has been especially dynamic over the past 40 years, with a rapid increase in the 1990s and a more recent decrease in fire area and frequency. New regional fire hotspots created by the Russian invasion into Ukraine and associated land abandonment add to the current dynamics [18].

Despite its global importance, changes in fire regimes across the Eurasian steppes and their ecosystem implications remain patchily studied and poorly synthesized. This is unfortunate as the area comprises a massive proportion of the worlds intact grasslands [19]; still holds extensive, low-input, socio-ecological pastoralist systems [20]; harbours many globally threatened and often endemic species [21]; provides opportunities for ecosystem-scale restoration and rewilding [22]; and contributes extensively to global food security [23].

We systematically reviewed the literature and data to answer three main research questions: (i) How have fire regimes changed across the Eurasian steppe since 1980? (ii) What were the drivers of observed changes? (iii) How do grassland fires, and changes in fire regimes, affect biodiversity and ecosystem properties?

## 2. Study region and methods

We constrained our review to the Eurasian steppes, grasslands stretching from eastern Ukraine to the Altai mountains characterized by frequent, intense and large fires ([3,14,24]; electronic supplementary material, figures S1–S4). Nearly all of the Ukrainian steppes had been converted to cropland by the nineteenth century; therefore, most fires now originate from stubble burning. In contrast, across Kazakhstan and the adjacent Russian steppes, large expanses of natural grasslands still exist, often bordered by secondary grasslands on abandoned cropland ([25]; electronic supplementary material, figure S5). Here, fires are much larger than in Ukraine. We excluded the Asian steppes in Siberia, Mongolia and China, because they differ strongly in climate and biotic composition [23]. In Mongolia and China, but not in the steppes of Russian Siberia, high livestock densities largely suppress fire [26,27]. We also included the semi-deserts [28] in European Russia and Kazakhstan, as these are part of the global fire hotspots [29] and form a gradual transition zone from steppe to desert vegetation in Central Asia ([23]; electronic supplementary material, figure S2). We here consider the period 1980–2023, because of especially rapid and abrupt land-use change on the steppes and perceivable climate change.

We conducted a systematic review following Foo *et al.* [30]. We formulated research questions, followed by a scoping search, literature mapping and a tailored, iterative, scoping-based literature search, including forward and backward searches. We also searched Russian-languages sources and reviewed data on land use (trends in arable land and livestock numbers) and fire patterns. For details on the methods see electronic supplementary material, text S1.

## 3. Fire regime change 1980–2020

**Figure 1.**
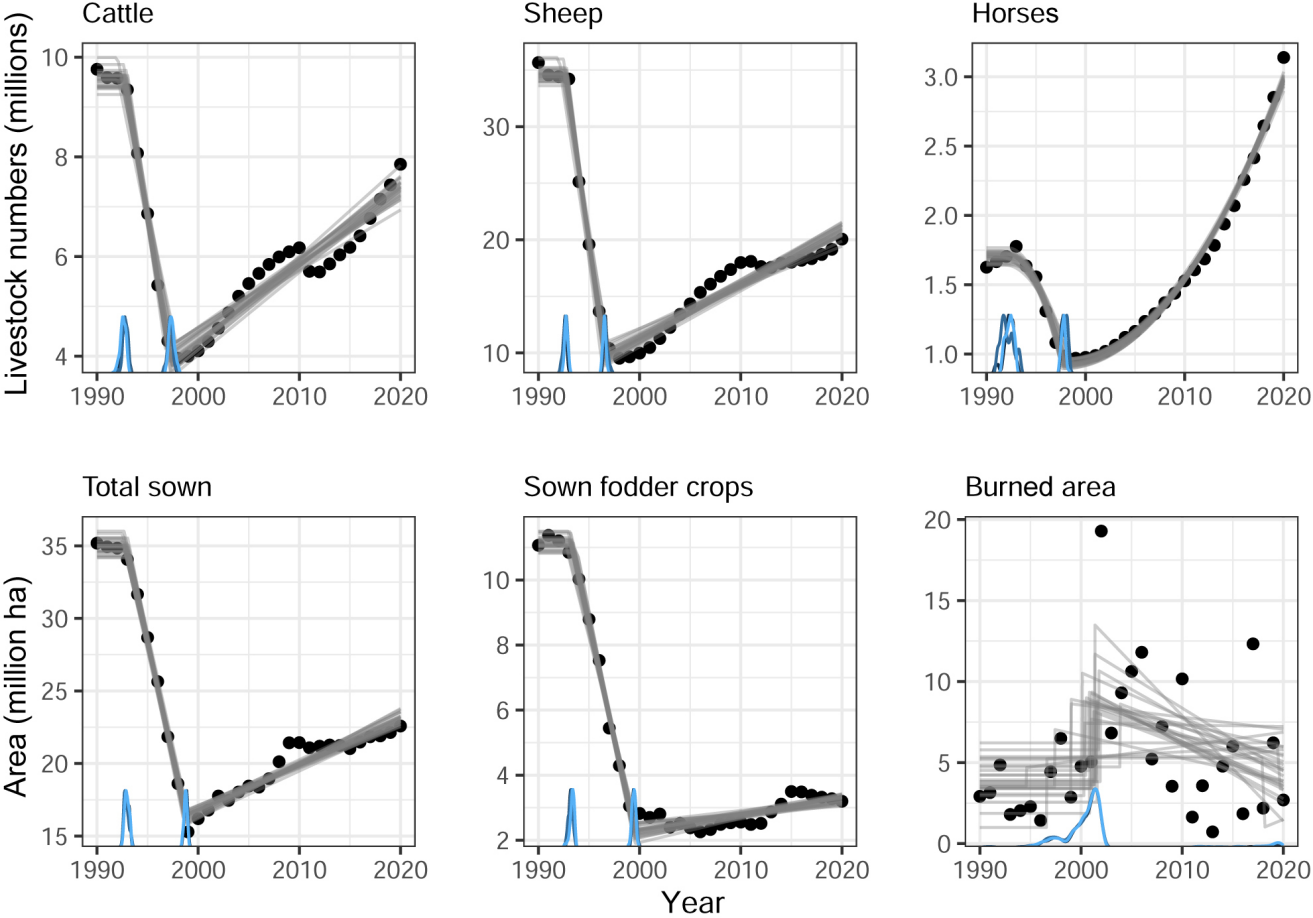
Livestock numbers and sown area across Kazakhstan, and mapped burned area across the forest steppe, steppe and semi-desert ecozones of Kazakhstan (based on AVHRR and MODIS remoting sensing products, 1990–2020). Black dots are raw data, grey lines are fitted partial regression lines drawn randomly from the posterior distribution (illustrating uncertainty in the predictions) and blue lines are the posterior density of estimated change points (cf. electronic supplementary material, text S1 for methods).

Fire regimes are characterized by fire size, fire interval (recurrence rate), seasonality, intensity and spread pattern (horizontal versus vertical, [31]). Fire area and fire size across the steppes of Kazakhstan were rather stable during the 1980s and until *ca* 1995 ([32], figure 1) but increased rapidly and abruptly in the late 1990s. A high-intensity regime persisted until *ca* 2005 (Kazakhstan) but burned area decreased again from 2005 to 2015 (figure 1). In the Russian steppes, the period of high fire intensity lasted until *ca* 2010 [17,33]. Global maps suggest an increase in burned area from 3.79 to 15.5 Mha^−1^ across the steppes in the late 1990s [34]. Regionally, increases in burned area in the mid-1990s were extreme. In the Russian dry Volga steppes, the area burned was virtually zero in the period 1985−1995 but increased dramatically to 20% of a 19 000 km² study area between 1996 and 2007 [35]. Fire return rates increased to every 2–3 years in some areas, and *ca* 30% of local hotspots burned every 5 years or more often [36]. Across northern Kazakhstan, the area burned increased sevenfold, and the number of fires eightfold between 1990 and 2000. Between 2000 and 2016, the number of fires declined slightly, but the very large size of fires remained stable, especially in steppe grassland [37]. In western Kazakhstan, fire frequency was 10 times higher in 2001−2010 compared with 1986−2000 [38,39]. In steppes of the Russian Altai, the number of fires and fire area continued to increase until 2014, with a decrease in fire interval [40].

The rapidly increasing trend in burned area was reversed around 2005 (figure 1). Between 2002 and 2016, the total area burned declined by 53% in northern Eurasia, with grassland fires accounting for 93% in this trend; Kazakhstan contributed 47% of the total area burned and 84% of the decline [24,27]. A decreasing trend in burned area was also observed on the meadow steppes of European, Russian and western Siberia in 2000−2021 [41].

Fire seasonality follows a bimodal pattern. In April and May, relatively small and localized fires occur in agricultural areas of the more productive steppes due to stubble burning [37,42]. From July to October, large fires of up to 500 000 ha burn especially in the steppes in the south of the study region, where the plant biomass is dry and highly flammable due to more arid climatic conditions [35,37,41]. These patterns are also traceable in the atmosphere from black carbon emissions, with a clear peak in May and two later peaks in August and October [43]. The decrease in fire area between 2003 and 2015 went along with decreasing black carbon emissions across the Eurasian steppe [43].

**Figure 2.**
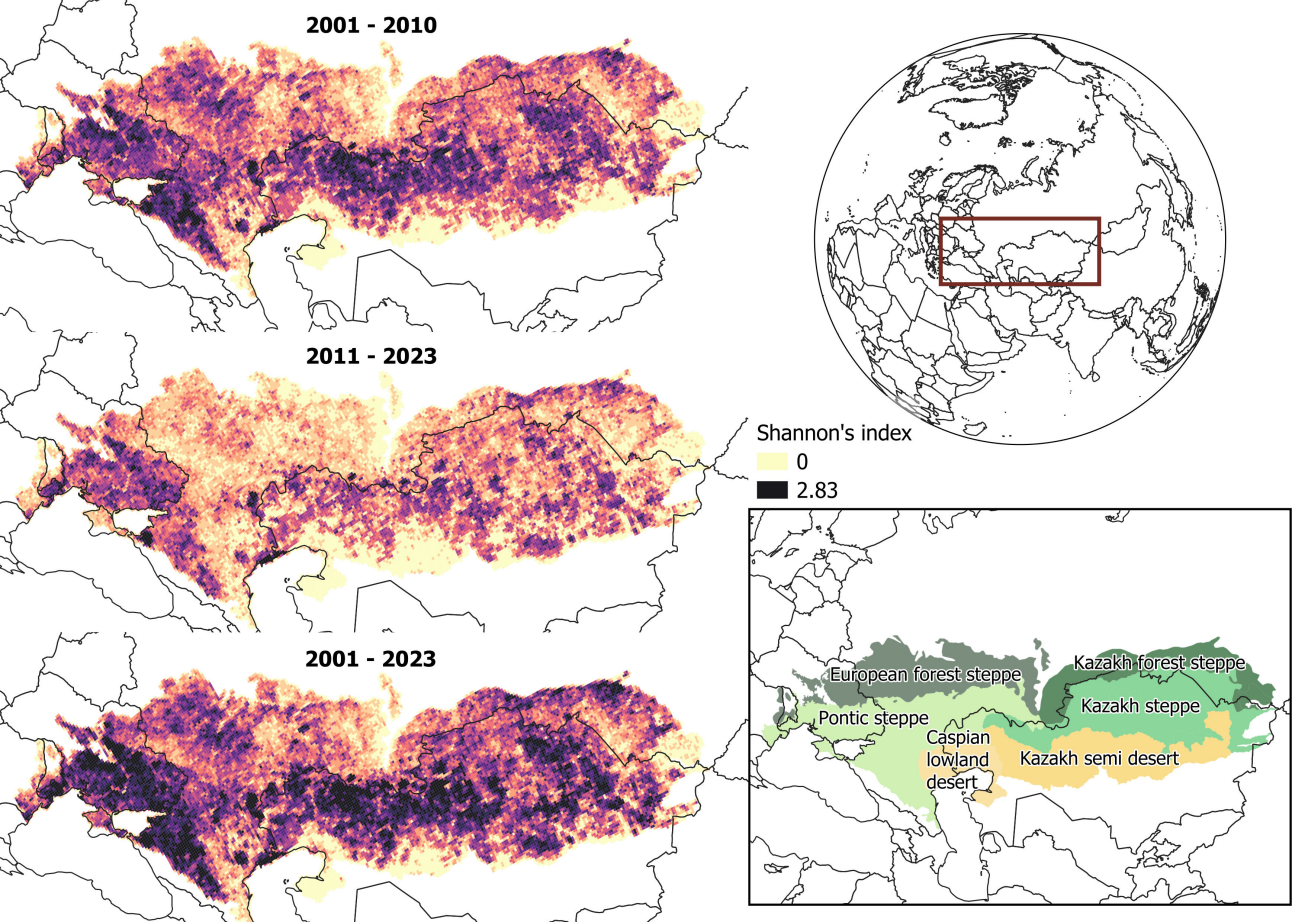
Pyrodiversity in 15 km × 15 km grid cells across the study region (inset, main ecozones after Olsen *et al.* [28]). Pyrodiversity is displayed as the Shannon diversity in fire frequency across all 500 × 500 m MODIS burned area product pixels per 15 km grid cell for the periods 2001−2010, 2011−2023 and 2001–2023. Darker colours indicate a higher diversity. Cf. electronic supplementary material, text S1 for methods.

The coupling of temporal and spatial changes in fire regimes (‘pyrodiversity’) can also change overall landscape diversity and heterogeneity, which drives biodiversity [44]. Changes have seldom been mapped over larger areas, but an analysis for the Western Palearctic suggested that the steppe biome had a much higher pyrodiversity than any other biome [45]. Pyrodiversity effects on ecosystem functioning and biodiversity have rarely been studied, especially outside North America and Australia, and in grasslands. Across our study area, pyrodiversity, measured as the Shannon diversity H´ in time since last fire across 15 km × 15 km grid cells (cf. electronic supplementary material, text S1), was 0.60 ± 0.003 s.e. across the study region in 2001–2010, a period with high fire disturbance. It declined to 0.38 ± 0.002 s.e. in 2011–2023, when fire disturbance was much lower (figure 2).

## 4. Drivers of changing fire regimes

### (a) Land-use change

**Figure 3.**
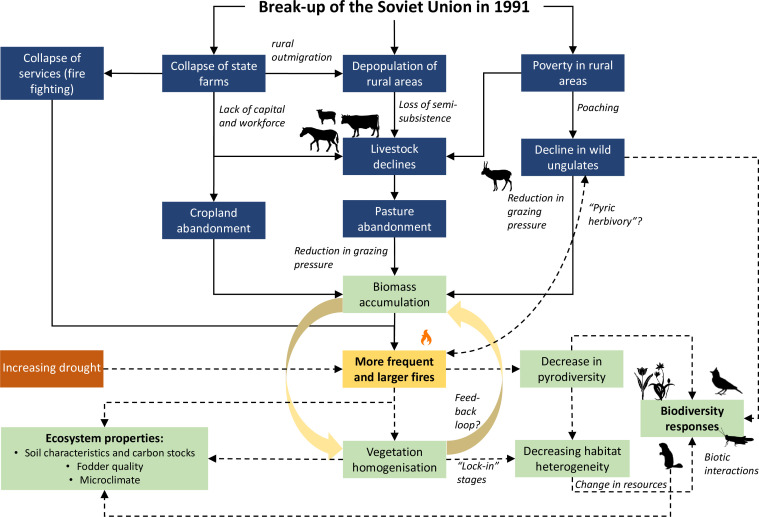
Processes, outcomes (boxes) and potential drivers (in italics) of increasing fire disturbance due to post-Soviet land abandonment between *ca* 1995 and 2005 that persist across parts of the Eurasian steppe until today. Solid lines connect patterns that are supported by published evidence. For those linked by dashed lines, evidence is either scarce or hypothetical. Boxes in blue summarize socio-economic processes and outcomes, those in green biodiversity responses and ecosystem responses.

The increase in fire disturbance during the 1990s was largely caused by land-use change following the break-up of the Soviet Union in 1991 [38,39,46]. The transition from a state-controlled, socialist, to a free market economy meant the collapse of state farms over the entire area of the Russian and Kazakh steppes in the early 1990s (figure 3). This loss of rural infrastructure and employment triggered massive rural outmigration [22]. Both processes resulted in the abandonment of 40−60 million ha cropland across the former Soviet Union [25]. At least 20 million ha (*ca* 50% of the Soviet-period cropland) were abandoned across the Kazakh steppes around the year 2000 (figure 1; electronic supplementary material, figure S5) and similar amounts across the Russian steppes, making the former Soviet Union a global hotspot of cropland abandonment [47].

Along with the crumbling state farms and rural outmigration, populations of domestic grazing livestock also collapsed ([20,48], figure 1) and left vast expanses of steppe pasture abandoned [46]. Widespread poverty during the 1990s increased poaching pressure on saiga antelope *Saiga tatarica*, leading to a population decline from >1 million in 1990 to a mere 30 000 animals [49] and a further reduction of grazing pressure on the steppe.

The increase in area burned and fire frequency in the early 2000s was attributed to a build-up of flammable biomass on abandoned pastures and cropland through vegetation succession: 4 –6 years after destocking, accumulated fuel increased fire frequency across the Russian steppes [42,50] and western Kazakhstan [38]. Low grazing probability was strongly associated with high fire disturbance and in the Kazakh steppes: field data suggested that where grazing intensity fell below a threshold of *ca* 250 dung piles ha^−1^, fire frequency would increase fivefold [51]. Firefighting systems deteriorated in the 1990s due to a lack of funding and lack of infrastructure after the collapse of the state farms [17]. Overall, a transition from a grazing-dominated to a fire-driven disturbance regime has been suggested for the 1990s [46,51].

The increase in fire area and fire return interval was reversed in the mid-2000s (figure 1), albeit not to Soviet-period levels. Total burned area decreased on the Eurasian steppes in the period 2002–2016 [27]. This was related to decreasing net primary productivity, likely due to increasing biomass consumption by recovering livestock numbers (figure 3; [27,52]). Across Kazakhstan, rapid declines in cattle, sheep and horse numbers as well as area sown set in 1992–1993, but were reversed 1997–1999, albeit slowly (figure 1). Horse numbers increased strongly from 1998 (figure 1), mirrored by a reactivation of horse pasture over large areas [53], but cattle and sheep numbers are still much lower than in Soviet times before 1990, which explains the delayed response of fire regimes [37]. In Russia, fire disturbance declined less and later, as livestock numbers have not recovered yet [33].

### (b) Climatic changes

Climate is an important determinant of fire regimes globally: increasing aridity and decreasing precipitation can lead to vegetation changes with more flammable biomass [7]. Increasing temperatures, often coupled with decreasing precipitation, increase ‘fire weather’, i.e. periods of a high probability of ignition.

In Kazakhstan, where the largest steppe fires occur (electronic supplementary material, figure S3), annual mean air temperature increased by *ca* 2°C with a strong increase in extreme temperatures in the period between 1941 and 2011, whereas there was only a weak decreasing trend in precipitation [54]. More recently, from 1980 to 2015, temperatures increased especially in the western part, whereas precipitation increased over most of Kazakhstan [55].

Across Kazakhstan, the total area burned per year was significantly negatively correlated with precipitation, soil moisture and relative humidity. It was positively correlated with the frequency of hot days during the burning season, from June to September [56]. These results stem from simple annual correlations. However, the study period, from 1997 to 2016, covers both the rise and decline of the intensive fire regime and might therefore hint to some climatic influence independent of land use. In the Russian steppes, in addition to a lack of grazing, the increase in fire area in the 1990s was correlated with high temperatures in August leading to drought and high availability of dry biomass, but associations were much weaker than those with grazing parameters [50].

For the period 2002–2016, there was a statistically significant interaction of climate and land use [27]: burned area decreased where precipitation increased, but this was moderated by livestock density. The area burned was unaffected by grazing intensity in dry years, but in wet years with high amounts of biomass, higher grazing intensity was associated with less burned area.

In summary, the steppe fire regime seems to have been fuel-limited in Soviet times due to higher livestock density, but became somewhat more sensitive to climate after the number of native and domestic ungulates collapsed in the early 1990s. Such patterns have also been observed elsewhere, e.g. in the western Mediterranean Basin [8].

## 5. Fire impacts on ecosystem properties

### (a) Vegetation and plant communities

The collapse of wild and domestic ungulate populations in the early 1990s resulted in the build-up of massive amounts of fuel across the Kazakh and Russian steppes (figure 3): tall-growing stands of highly flammable grasses developed, together with thick litter layers, a condition that allows ignition and rapid fire spread [57,58]. The resulting increase in fire size and frequency on abandoned pasture and cropland led to a decline of annuals, woody forbs and dwarf shrubs (especially *Artemisia* spp., [51]). Vast, homogenous expanses of grass developed, dominated by often only one or two species, especially *Stipa lessingiana, Stipa sareptana, Stipa capillata, Agropyron cristatum* and *Leymus ramosus*, peaking in the early 2000s when the fire regime was most intense. Grasses recover quickly on burned areas in comparison to forbs, due to increased post-fire nutrient mobilization [59], and litter accumulates within a few years [60]. The cycle of fast grass and litter recovery, new ignition and vegetation homogenization has likely led to a feedback loop on the steppes that allows large, grassy areas to persist [61].

Grass encroachment, structural and functional simplification and declining plant species richness at high fire frequency were also observed across the western Eurasian steppes, e.g. in Crimea [62], the Volga area [63,64] and the southern Ural steppes [60,65]. Grass encroachment suppresses the establishment of woody plants in the forest steppe [64,66].

The resistance, resilience and temporal stability of vegetation structure and plant community composition at different fire frequencies have not been studied extensively. On abandoned cropland restoring back into steppe, in the absence of grazing and following grass encroachment, fire might delay restoration to steppe plant communities [67]. In the more productive Ukrainian steppes, the cover of vascular plants recovered within a year, whereas the cover of mosses and plant litter remained sparse for 4 years [68].

In the dwarf shrub-dominated semi-deserts of Kazakhstan, recovery trajectories seem to differ from the grassy steppes: species richness and α-diversity of plants were twice as high in the first 3 years after fire compared with controls that had not burned for at least 35 years but declined again from year 8 to 30 [69]. Here, fire disturbance creates vegetation heterogeneity, as a dense, closed sagebrush canopy is removed by fire, allowing for colonization of annuals and seed germination from the soil seed bank [69].

### (b) Soil properties, carbon stocks and biomass

The change from a grazing-dominated to a fire-driven regime had likely important implications also for soil, biomass and carbon stocks. Fire promotes bare ground, resulting in an increase in topsoil temperature amplitude and transpiration. Soil moisture is reduced during the first 3 years after steppe fires [70,71], e.g. up to 30% in the 0−30 cm soil layer in the Orenburg region of Russia [72]. This affects the movement and chemistry of soil solutions: soluble salts, carbonates and sulfate concentrations increase in the upper layer of the soil profile, with phosphorus and potassium contents returning to the level at control sites 3 years after a steppe fire [72]. Grass litter burns almost completely, but restores 3−4 years after fire [73]. Similar recovery times in soil characteristics, i.e. humus, black carbon, pH and soluble salts, were found in the dry steppe of southern Ukraine [74]. In the Trans-Urals area of Russia, fire effects were restricted to the uppermost horizon of 0−5 cm soil depth, where soil pH, organic carbon, nitrogen, plant available phosphorous and potassium and exchangeable base cations such as sodium and calcium increased [59]. These effects were still visible 2 years after fire. Enzyme activity declined after fire but restored within less than a year. In summary, fire leads to short-term fertilization effects, allowing fast and dense regrowth of grasses.

Regular fires cause gaseous losses of carbon and nitrogen, with an estimated global average carbon loss of 18% under repeated grassland fires [75]. For the western Eurasian steppe belt, the recent decline in fire disturbance was predicted to result in carbon gains [76]. However, combustion-related carbon losses can partly be offset by the accumulation of pyrogenic carbon, with 8–17% in Chernozems and Kastanozems of southern Russia [77]. The highest concentrations of pyrogenic carbon are found in a depth of over 50 cm, illustrating the important role of social, burrowing rodents that move carbon from the surface (where it is sensitive to combustion during subsequent fire [78]), to deeper soil layers. Direct carbon losses, accumulation of black carbon and changes in bioturbation due to fire-induced declines of burrowing mammals (see §6a) will have influenced the carbon cycle on the steppes over the past 40 years, but the contributions of each effect remain largely unstudied.

### (c) Fodder quality

Feather grass *Stipa* spp. has a high fodder value in spring when leaves exhibit a high palatability and high nutrient contents. From June onwards, the nutritional value rapidly decreases due to high lignin and leaf dry matter content [51,79] and the allocation of leaf nutrients such as N and P to the root system. The dominance of *Stipa* over large areas in the 2000s has likely reduced fodder quality, also because annual and biennial species declined where *Stipa* expanded [67]. As these ruderals have a high specific leaf area and high seed and leaf nutrient contents, they have a high palatability and fodder quality. The currently observed recovery in wild and domestic ungulates (see §4a) might, therefore, increase fodder quality in previously ungrazed areas, at least under moderate stocking densities.

## 6. Fire impacts on faunal biodiversity

### (a) Vertebrates

Small, social, herbivorous and burrowing mammals play an important role as ecosystem engineers in most grassland systems of the world [80]. A high fire frequency between 2000 and 2015 had a negative effect on the occurrence and abundance of bobak marmot *Marmota bobak* [81] and yellow-ground squirrel *Spermophilus fulvus* [79], and the latter species recovered in population density with progressing time after fire. The dense, homogenous vegetation dominated by grasses that dominate in areas of frequent fire (cf. §5a) make movement difficult, decrease fodder palatability and plant nutrient contents and obstruct views, which increases predation risk e.g. by steppe eagles *Aquila nipalensis* and foxes [79]. In mid-day gerbils *Meriones meridianus*, the effects of pasture abandonment, including a fire-triggered shift to grass dominance, led to a population crash [82]. In birds, species richness and abundance were negatively related to high fire frequency, fire area and extent in 2009–2017 on moderately and ungrazed steppe, i.e. the steppe types with the highest fire frequency. There were more species with decreased abundance than those reaching higher abundances at high levels of fire disturbance. Fire legacy effects were detectable for at least 8 years [32]. In contrast, bird responses were positively related to high-burn frequency in semi-deserts [32], as birds here might have profited from a higher heterogeneity in vegetation where fires occur [69]. Mechanisms behind negative responses to high fire frequency include habitat loss, e.g. in the globally threatened great bustard *Otis tarda* in the Russian forest steppe [83], and burning of nests in the globally threatened steppe eagle *Aquila nipalensis* as well as declines in small mammals, their main prey [84].

### (b) Invertebrates

The post-Soviet increase in fire disturbance was associated with a deline of specialist steppe invertebrate species and a reorganization of communities [42]: in the Russian steppes, the abundance of tenebrionid and carabid beetles did not change with increasing fire disturbance, but specialist species were replaced by generalists. The abundance of curculionid beetles, Heteroptera, spiders, woodlice and myriapods showed a strong decrease directly after fire suggesting negative effects of high fire frequency. An increase in fire size meant that there were less refugia left for these taxa in the period of frequent burning [42]. In meadow steppes of Russia, richness and activity density of ground-dwelling beetle species increased in the second post-fire year, while that of spiders decreased [85]. A comparison of burned and unburned steppe in eastern Ukraine suggested a decrease in abundance, but an increase in richness in spider communities immediately after fire, but also identified species that occur exclusively on burned sites. In the year after fire, diversity differences disappeared, but abundances remained lower at burned sites [86]. Where fire intensity increased in the post-Soviet period, species richness decreased threefold after fire but had recovered almost entirely after 3 years. Fire effects were positive for species preferring bare ground, but negative for litter dwellers and rare, range-restricted species [87]. After a recovery period of 5 years, spider communities had largely reached pre-fire abundance, diversity and community composition, whereas abundance and diversity of ground-dwelling beetle communities remained significantly different from pre-fire communities with regard to trophic guild ratios and dominance structures [68].

In summary, intensifying fire regimes have led to a taxonomic, functional and structural homogenization of plant communities and vegetation. Animal communities at lower trophic levels (insects and spiders) seem to be impacted directly by fire, but often recover quickly, although changes in community composition occur. Small mammals and birds seem to suffer from intensifying fire regimes indirectly through vegetation homogenization. However, responses are often species specific and the available evidence is rather scarce, generalization is therefore difficult.

## 7. Discussion

We provide evidence that a period of extremely rapid decline in wild and domestic ungulates, caused by the collapse of the Soviet Union, triggered a regime shift towards a strong increase in fire disturbance on the Eurasian steppe during the second half of the 1990s, that persisted for at least 10 years until recovery in livestock and increasing grazing pressure started controlling fire again. Fire regimes were additionally affected by increasing aridity and changes in precipitation, but likely only after changing land use allowed fuel to accumulate. This shift had largely negative consequences for ecosystem functioning, with vegetation homogenization, biodiversity loss and community reorganization. We here outline important research gaps that our review identified and suggest future research directions.

### (a) Baselines: historical fire regimes

Current fire management should operate from baselines informed by historical fire regimes [88]. Paleoecological evidence suggests that fire has been an integral part of the Mongolian steppes since the early Holocene [15], but a lack of charcoal remains points to fire suppression by a long grazing history, dating back at least to 1200, perhaps to 6000 years BP [89]. Holocene fire disturbance levels are less clear for the Eurasian steppes, as the global charcoal database does not contain data from this ecoregion [15]. Charcoal concentrations from the Russian forest steppe suggest a high fire frequency *ca* 13 000–8000 years BP [90]. In the mountain steppes of Armenia, high charcoal concentrations coincided with amounts of grass pollen, while low charcoal concentrations were associated with high Chenopodiaceae pollen concentrations [91]. Chenopodiaceae indicate grazed (and therefore fire-suppressed) steppe. A decline in Poaceae and an increase in Chenopodiaceae pollen since 4200 years BP [91] could point to early fire suppression, coinciding with the development of animal husbandry and mobile pastoralism on the Eurasian steppe [92]. However, periods of abundant pollen grazing indicators and high charcoal concentrations overlapped in the Russian forest steppe [90].

In the eigteenth and nineetenth centuries, anecdotal evidence from narrative accounts of explorers and ethnographs suggests that fire was used by Kazakh, Nogai and Kalmyk tribes for pasture management and tactical warfare across the Eurasian steppes [17]. Reports from the 1920s indicate that fire intensity increased during the period of Stalin’s collectivization and sedentarization of nomads, with associated famine and massive human outmigration to Mongolia and China [20]. This caused a collapse in livestock numbers in the same order as the post-Soviet changes we describe here [20], but the consequences for fire regimes are unknown. The later Soviet period between 1950 and 1980 was characterized by low fire frequency and small fire size due to strict fire suppression and high grazing levels [17].

Taken together, fire regimes of the Eurasian steppes over the past centuries, but also on geological time scales, seem understudied. More research on charcoal deposition seems necessary to establish fire intensity baselines. The results could then serve to model the effects of different fire regimes on the steppe ecosystem.

### (b) Understanding fire–grazing interactions for restoration and rewilding

Across the steppes of Russia and Kazakhstan, millions of hectares of cropland and pasture remain abandoned [25]. For vast areas, it is still unclear when and to what extent land use will return due to a lack of infrastructure and ongoing human outmigration [22]. Here, considerable potential for ecosystem restoration and rewilding exists [22]. Currently, ‘passive rewilding’ [93], i.e. the absence of human restoration interventions, prevails on abandoned land. We have shown here that the continued lack of grazing and the associated intensification of fire regimes has likely had negative ecosystem consequences. It might, therefore, be more desirable to follow two potential pathways to rewilding: (i) a reintroduction of large herbivores to their native range with management tapering out over time (‘active rewilding’ *sensu* [93], (ii) an expansion of traditional livestock grazing systems into abandoned pasture areas that mirror grazing intensity and patterns of native grazers (partial restoration).

Large efforts towards active rewilding are already being made in Kazakhstan. Saiga antelopes have recovered from an all-time low of *ca* 30 000 animals in 2002 to a population of *ca* 1.9 million in 2023 due to better protection [94]. Depleted and extinct large herbivores such as kulan *Equus hemionus* and Przewalski’s horse *Equus przewalski* are reintroduced [95]. In contrast, the future of free-ranging livestock production is unclear across the steppes, and livestock systems might intensify, with free-ranging grazing systems transitioning to feedlot-based production [46].

It seems timely to study the implications of these various strategies in an integrated fire management approach: assessing detrimental and benign effects of fire, weighting benefits and risks, and responding appropriately and effectively based on predefined objectives [96]. Acknowledging the strong fire–grazing interaction in the system, this could be implemented through the lens of ‘pyric herbivory’ [12]: herbivores prefer nutrient-rich and palatable biomass, which is available after a certain time has passed after fire. Animals move to find patches with optimal foraging conditions, and, through their grazing, affect the build-up of new biomass, which in turn affects ignition probability.

Land management strategies should therefore (i) quantify the scale and spatio-temporal pattern (incl. pyrodiversity, see §3 above) of pyric herbivory with the help of remote-sensing data on fire and tracking data on wild and domestic herbivores; (ii) quantify ecosystem resilience, i.e. determine recovery times and baselines, quantify potential feedback loops (see §5a) and establish grazing levels to halt these; (iii) develop scenarios of varying fire frequencies, grazing intensity levels and their interactions that maximize biodiversity, ecosystem functions and fodder quality for livestock; and (iv) integrate Protected Areas into rewilding strategies as a strict prohibition of grazing has led to increased fire activity [97].

### (c) Climate change impacts on fire regimes

Our review suggests that climate change has been affecting fire regimes on the Eurasian steppe, but likely only after fuel accumulation due to declining grazing pressure. Several future scenarios seem plausible: a further recovery of wild and domestic grazers would reduce fuel loads, therefore minimizing the impact of climate on fire regimes. In contrast, a stagnation of agricultural reclamation would mean a continued high load of flammable biomass on abandoned cropland and pasture.

A stronger-than-global-mean-warming trend is predicted for Central Asia until the end of the century, with increasing drought in summer but also increasing spring and summer precipitation [98]. Drought events are predicted to become more frequent, with longer duration, higher severity and intensity [99]. Current and future climate change patterns are highly variable across the steppes [55]. This already translates into variable regional change in vegetation cover, the area of green and the area of photosynthetically active vegetation [100]. Given this large number of potential levers of biomass availability, flammable vegetation development and fire weather, predictions of future fire regimes are difficult: for the Eurasian steppes, they range from an expert-based likelihood of <50% of *any* regime shift by 2100 [4] to a prediction of 3–13% increase in burned area by 2080 [101].

Adaptive fire management on the steppes would, therefore, benefit from the consideration of interacting climate change and land-use scenarios, and more regionalized efforts to model the effects of predicted climate change. Ideally, this would also be combined with a spatial assessment of ignition risk: most fires in the area are of anthropogenic origin [17,42], and this is driven by socio-economic developments such as recolonization of recultivated areas, or continued outmigration.

### (d) Armed conflict

In addition to sociopolitical shocks such as the collapse of the Soviet Union, warfare and armed conflict can affect land-use decisions and trajectories [102,103]. The exact consequences of conflict for land use [104], fire regimes and biodiversity [105] are poorly understood, but there is evidence that warfare impact overruled climate in controlling fire regimes along the Silk Road in China and Central Asia over the past 2000 years [106]. The Russian war against Ukraine since February 2022 has resulted in an increase in fires, and warfare concentrates in the steppe ecozone. War-related land abandonment has affected *ca* 8% of the cropland in 2022 alone [107], leading to biomass accumulation and providing fuel for fire. Ignition happens through shelling and artillery combat. In 2023, over 40% of the fires were on grassland (including abandoned cropland), mostly in the steppe ecozone [108]. In July 2022, more than 100 000 ha were burning [18], including repeated, large fires in Protected Areas in the steppe zone. In contrast to Kazakhstan and parts of Russia, only tiny, fragmented steppe grasslands remain in Ukraine due to widespread transformation into cropland (electronic supplementary material, figure S5). The change in fire regimes in natural grasslands will, therefore, have a disproportionately large effect on ecosystems and biodiversity at the westernmost part of the steppe zone, which merits more research on potential consequences. This is even more important as the war effects will add to the long-term legacies of the collapse of the Soviet Union, and therefore provide a perspective on linked, legacy-driven fire disturbances.

## Data Availability

All bibliographic data, livestock and fire data as well as Google Earth Engine and R-scripts to reproduce the analyses and figures are available on Zenodo [109]. Supplementary material is available online [110].
